# Supplementary figures and images for: Genomic alterations involved in fluoroquinolone resistance development in Staphylococcus aureus
Source: PLoS One. 2023 Jul 26;18(7):e0287973. doi: 10.1371/journal.pone.0287973 (PMC10370734; doi:10.1371/journal.pone.0287973)

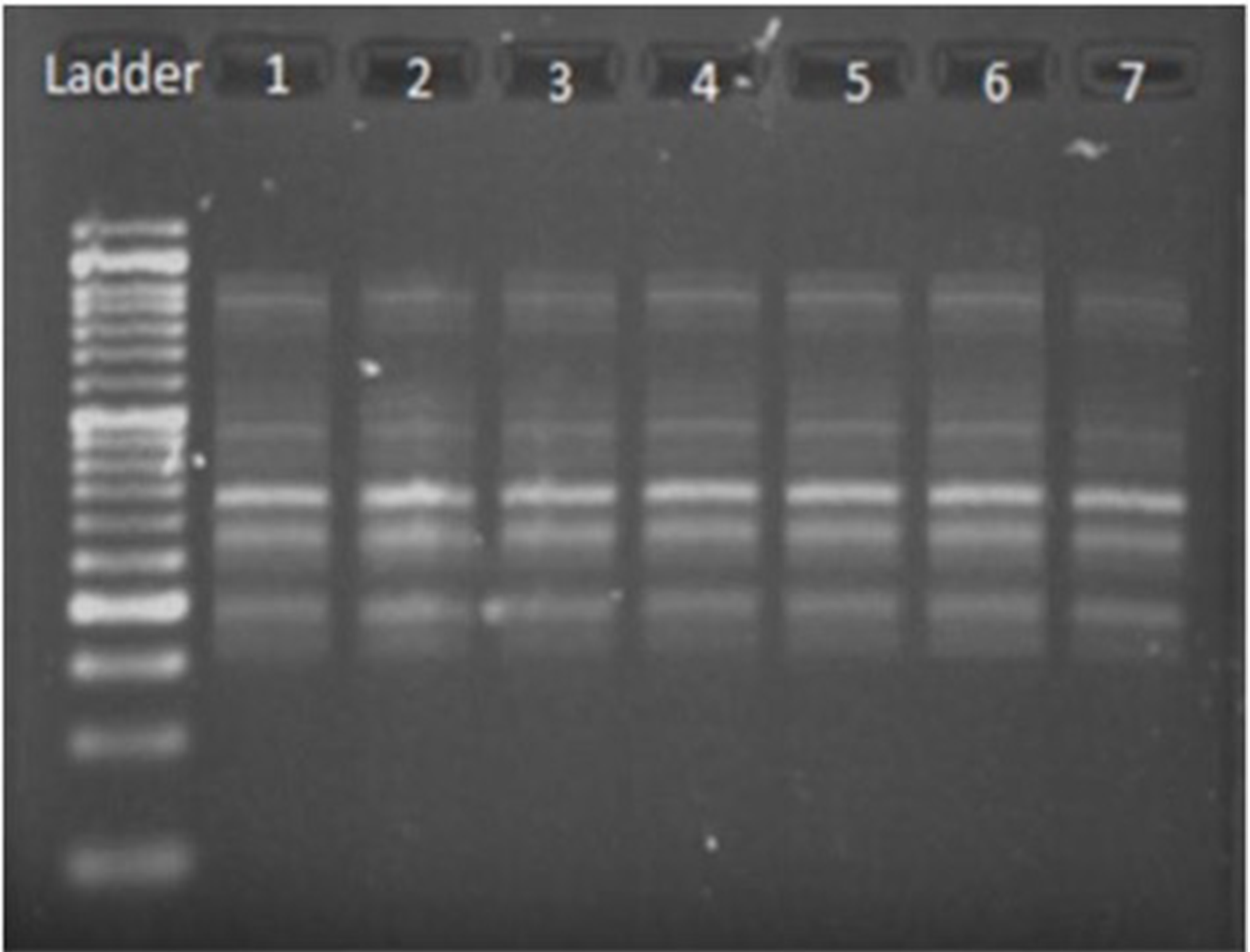

Supplement: S1 Fig — Ladder 100 bps; 1, S. aureus ATCC 29213; 2, CIP-1; 3, CIP-2; 4, LEV-1; 5, LEV-2; 6, OFL-1; 7, OFL-2. (TIF) [file pone.0287973.s001.tif]
