# Supplementary material for: Genomic alterations involved in fluoroquinolone resistance development in Staphylococcus aureus
Source: PLoS One. 2023 Jul 26;18(7):e0287973. doi: 10.1371/journal.pone.0287973 (PMC10370734; doi:10.1371/journal.pone.0287973)
Supplement: S1 Table — (DOCX) [file pone.0287973.s002.docx]

S1 Table. The effect of reserpine on antibiotic susceptibility profile of initial and *S. aureus*-1 strains.

|  | **MIC (mg/L)** | | | | | | | |
| --- | --- | --- | --- | --- | --- | --- | --- | --- |
|  | **Initial *S. aureus*** | | **CIP-1** | | **LEV-1** | | **OFL-1** | |
|  |  | **With reserpine** |  | **With reserpine** |  | **With reserpine** |  | **With reserpine** |
| Ciprofloxacin | 0.25 | 0.25 | 8 | 8 | 4 | 4 | 8 | 8 |
| Levofloxacin | 0.125 | 0.125 | 4 | 4 | 8 | 4 | 4 | 2 |
| Ofloxacin | 0.25 | 0.25 | 4 | 2 | 8 | 8 | 16 | 16 |
| Moxifloxacin | 0.0625 | 0.0625 | 0.25 | 0.25 | 0.5 | 0.25 | **0.5** | **0.125** |
| Nalidixic acid | 16 | 8 | 128 | 128 | 128 | 64 | 128 | 128 |
| Ampicillin | 16 | 16 | 256 | 256 | 128 | 128 | **128** | **34** |
| Amoxicillin | 0.5 | 0.5 | **32** | **8** | 64 | 64 | **64** | **16** |
| Cefalexin | 0.5 | 0.5 | 4 | 4 | 8 | 8 | 64 | 32 |
| Chloramphenicol | 16 | 8 | 32 | 32 | 32 | 32 | 16 | 16 |
| Doxycycline | 0.125 | 0.125 | **4** | **1** | 2 | 2 | **4** | **1** |
| Erythromycin | 0.25 | 0.25 | **32** | **8** | 32 | 16 | 16 | 16 |
| Lincomycin | 0.5 | 0.25 | 32 | 16 | **128** | **32** | **64** | **16** |
| Oxacillin | 0.5 | 0.25 | 16 | 16 | 32 | 32 | 8 | 8 |
| Tetracycline | 8 | 8 | 16 | 16 | 8 | 8 | 16 | 16 |
