# Supplementary material for: Genomic alterations involved in fluoroquinolone resistance development in Staphylococcus aureus
Source: PLoS One. 2023 Jul 26;18(7):e0287973. doi: 10.1371/journal.pone.0287973 (PMC10370734; doi:10.1371/journal.pone.0287973)
Supplement: S2 Table — (DOCX) [file pone.0287973.s003.docx]

S2 Table. List of single nucleotide polymorphisms (SNPs), variants, and amino acid changes found in *S. aureus*-1 (CIP-1, OFL-1, and LEV-1) and *S. aureus*-2 (CIP-2, OFL-2, and LEV-2) strains but not in *S. aureus* ATCC 29213.

| **SNP/variant and GenBank Accession** | **Amino acid changes** | **Systematic and trivial gene name** | **Protein encoded** | **Mutations in Samples** | | | | | | |
| --- | --- | --- | --- | --- | --- | --- | --- | --- | --- | --- |
|  |  |  |  | ***S. aureus* ATCC 29213** | **CIP-1** | **OFL-1** | **LEV-1** | **CIP-2** | **OFL-2** | **LEV-2** |
| A31132G MOPB01000016.1 | V110A | BJI72_0995  isdB | Heme transporter IsdB | √ | √ | √ | √ | √ | √ | √ |
| T35301A MOPB01000017.1 | I51N | BJI72_1046  mraZ | Transcriptional regulator MraZ | √ | √ | √ | √ | √ | √ | √ |
| C27139T  MOPB01000033.1 | No annotated protein | - | - | √ | √ | √ | √ | √ | √ | √ |
| A47198ATAT  MOPB01000036.1 | I19_I20insI | BJI72_1908  atpA | ATP synthase protein I | √ | √ | √ | √ | √ | √ | √ |
| T30604A  MOPB01000042.1 | I147_insR | BJI72_2151  LytT | LytTR family transcriptional regulator | √ | √ | √ | √ | √ | √ | √ |
| A338G  MOPB01000051.1 | Synonymous  mutation | BJI72_2614  sasG | surface protein G | √ | √ | √ | √ | √ | √ | √ |
| A1022G  MOPB01000063.1 | E213G | BJI72_2645  sasG | surface protein G | √ | √ | √ | √ | √ | √ | √ |
| A1053G  MOPB01000063.1 | Synonymous  mutation | BJI72_2645  sasG | surface protein G | √ | √ | √ | √ | √ | √ | √ |
| A1161G  MOPB01000063.1 | Synonymous  mutation | BJI72_2645  sasG | surface protein G | √ | √ | √ | √ | √ | √ | √ |
| T903C MOPB01000063.1 | Synonymous  mutation | BJI72_2645  sasG | surface protein G | √ | √ | √ | √ | √ | √ | √ |
| G5009GAAC  MOPB01000035.1 | S33_S34insC | BJI72_1850  *rimI* | Alanine acetyltransferase | . | √ | √ | √ | √ | √ | √ |
| TATCTAGATGATG4318T  MOPB01000016.1 | G309_T315delinsC | BJI72_0480  SACOL0573 | PIN domain containing protein | . | √ | √ | √ | √ | √ | √ |
| A13766G  MOPB01000012.1 | No annotated protein | - | - | . | √ | √ | √ | √ | √ | √ |
| A13775T  MOPB01000012.1 | No annotated protein | - | - | . | √ | √ | √ | √ | √ | √ |
| A13777C  MOPB01000012.1 | No annotated protein | - | - | . | √ | √ | √ | √ | √ | √ |
| A13778T  MOPB01000012.1 | No annotated protein | - | - | . | √ | √ | √ | √ | √ | √ |
| T13781A  MOPB01000012.1 | No annotated protein | - | - | . | √ | √ | √ | √ | √ | √ |
| T13785G  MOPB01000012.1 | No annotated protein | - | - | . | √ | √ | √ | √ | √ | √ |
| CG13787C  MOPB01000012.1 | No annotated protein | - | - | . | √ | √ | √ | √ | √ | √ |
| C32411A  MOPB01000008.1 | Synonymous  mutation | BJI72_0464  hslO | heat shock protein Hsp33 | . | √ | √ | √ | √ | √ | √ |
| A179T  MOPB01000035.1 | E59D | BJI72_1847  FhuC | ATP-binding ABC transporter | √ | √ | √ | . | √ | √ | . |
| G104T  MOPB01000042.1 | No annotated protein | - | - | . | - | √ | . | . | . | . |
| T105C  MOPB01000042.1 | No annotated protein | - | - | . | - | √ | . | . | √ | . |
| G501A MOPB01000051.1 | A39T | BJI72_2614  sasG | surface protein G | √ | - | . | . | . | . | . |
| T238A  MOPB01000054.1 | Synonymous mutation | BJI72_2622  lpl5nm | tandem lipoprotein | . | - | . | . | . | . | √ |
| T247A  MOPB01000054.1 | Synonymous mutation | BJI72_2622  lpl5nm | tandem lipoprotein | . | √ | . | . | . | . | √ |
| A250C  MOPB01000054.1 | Synonymous mutation | BJI72_2622  lpl5nm | tandem lipoprotein | √ | √ | √ | . | √ | √ | √ |
| G964A  MOPB01000063.1 | E194K | BJI72_2645  sasG | surface protein G | √ | √ | √ | . | √ | . | √ |
| T1129G  MOPB01000081.1 | No annotated protein | - | - | √ | - | √ | . | . | . | √ |
| C1132A  MOPB01000081.1 | No annotated protein | - | - | √ | - | √ | . | . | . | . |
| CATAGGCTTGTT22457C  MOPB01000021.1 | N3181delinsR | BJI72_1235  Ebh | Hyperosmolarity resistance protein Ebh | . | . | √ | √ | . | √ | √ |
| T86957C  MOPB01000021.1 | Synonymous  mutation | BJI72_1284  lpl | lipoprotein | . | . | √ | √ | . | √ | √ |
| CTT26104C  MOPB01000037.1 | K1283delinsRG | BJI72_1954  *fmtB* | methicillin resistance protein FmtB | . | . | √ | √ | . | √ | √ |
| C93466T  MOPB01000004.1 | A318T | BJI72_0139  ThlA | acetyl-CoA acetyltransferase | . | √ | . | . | √ | . | . |
| G308933A  MOPB01000004.1 | R570H | BJI72_0342  *grlA* | DNA topoisomerase IV subunit A | . | √ | √ | √ | √ | √ | √ |
| TTATTATTCTGATGCTCATTATGTTATTTTGGATTTTTATGAGTTTTATTTTCCAGAAATTATTATTTCCAC332967T  MOPB01000004.1 | F12delinsEK | BJI72_0362 | hypothetical protein | . | . | √ | . | . | . | . |
| T13964TTA  MOPB01000008.1 | V69delinsIRINQ | BJI72_0448  purR | pur operon repressor | . | . | . | . | . | √ | √ |
| G52A  MOPB01000047.1 | Synonymous mutation | BJI72_2594  sdrE | serine-aspartate repeat-containing protein E | . | √ | √ | √ | √ | √ | . |
| C112602G  MOPB01000011.1 | A31P | BJI72_0631 | YaiI/YqxD family protein | . | √ | . | . | . | . | . |
| A17477T  MOPB01000024.1 | F89Y | BJI72_1372  dgkA | undecaprenol kinase | . | √ | . | . | . | . | . |
| GT177023G  MOPB01000014.1 | P265_I272delinsSSRSRI | BJI72_0928  qoxA | cytochrome c oxidase polypeptide II | . | . | √ | . | . | . | . |

√, the mutation present; ., no mutation; –, no coverage.
