# Supplementary material for: Genomic alterations involved in fluoroquinolone resistance development in Staphylococcus aureus
Source: PLoS One. 2023 Jul 26;18(7):e0287973. doi: 10.1371/journal.pone.0287973 (PMC10370734; doi:10.1371/journal.pone.0287973)
Supplement: S3 Table — (DOCX) [file pone.0287973.s004.docx]

S3 Table. The statistical analysis of fold change in gene expression between initial *S. aureus* strain and FQ-exposed *S. aureus* strains (mean ± standard error).

| **Genes** | ***S. aureus* trains** | **Fold change**  **(2^-^ ^ΔΔCt^)** | **CI 95%** | **p-value^a^** |
| --- | --- | --- | --- | --- |
| *sigB* | CIP-1 | 1.67 | (0.03, 0.03) | 0.000 |
|  | CIP-2 | 1.16 | (0.03, 0.03) | 0.007 |
|  | OFL-1 | 1.50 | (0.22, 0.24) | 0.036 |
|  | OFL-2 | 0.41 | (0.1, 0.11) | 0.021 |
|  | LEV-1 | 2.39 | (0.98, 1.22) | **0.077** |
|  | LEV-2 | 0.59 | (0.23, 0.28) | **0.125** |
| *sigS* | CIP-1 | 2.62 | (0.74, 0.93) | 0.014 |
|  | CIP-2 | 1.70 | (0.23, 0.26) | 0.010 |
|  | OFL-1 | 1.92 | (0.15, 0.15) | 0.002 |
|  | OFL-2 | 0.55 | (0.04, 0.05) | 0.002 |
|  | LEV-1 | 3.80 | (1.34, 1.8) | 0.013 |
|  | LEV-2 | 0.77 | (0.19, 0.12) | **0.112** |
| *mgrA* | CIP-1 | 4.51 | (1.74, 2.19) | 0.023 |
|  | CIP-2 | 2.34 | (0.45, 0.5) | 0.015 |
|  | OFL-1 | 3.29 | (0.68, 0.75) | 0.009 |
|  | OFL-2 | 2.48 | (0.58, 0.65) | 0.020 |
|  | LEV-1 | 12.63 | (2.57, 2.84) | 0.002 |
|  | LEV-2 | 4.71 | (1.25, 1.44) | 0.009 |
| *norA* | CIP-1 | 57.61 | (19.46, 23.35) | 0.002 |
|  | CIP-2 | 70.78 | (26.93, 33.59) | 0.003 |
|  | OFL-1 | 5.45 | (1.67, 1.95) | 0.010 |
|  | OFL-2 | 2.78 | (0.15, 0.16) | 0.001 |
|  | LEV-1 | 21.33 | (12.4, 16.84) | 0.016 |
|  | LEV-2 | 5.96 | (2.45, 3.11) | 0.019 |
| *norB* | CIP-1 | 6.87 | (3.51, 4.82) | 0.029 |
|  | CIP-2 | 3.11 | (0.83, 0.95) | 0.017 |
|  | OFL-1 | 5.77 | (2.68, 3.39) | 0.026 |
|  | OFL-2 | 2.46 | (0.3, 0.32) | 0.005 |
|  | LEV-1 | 15.66 | (5.67, 6.97) | 0.006 |
|  | LEV-2 | 5.81 | (1.93, 2.32) | 0.012 |
| *norC* | CIP-1 | 6.83 | (2.2, 3.49) | 0.011 |
|  | CIP-2 | 3.28 | (0.19, 0.69) | 0.003 |
|  | OFL-1 | 4.61 | (1.64, 4.88) | 0.030 |
|  | OFL-2 | 2.97 | (0.69, 0.68) | 0.013 |
|  | LEV-1 | 20.42 | (3.9, 4.57) | 0.001 |
|  | LEV-2 | 6.33 | (2.22, 3.52) | 0.014 |
| *rimI* | CIP-1 | 7.85 | (0.99, 1.05) | 0.001 |
|  | CIP-2 | 4.02 | (0.83, 0.92) | 0.007 |
|  | OFL-1 | 6.54 | (2.27, 2.71) | 0.011 |
|  | OFL-2 | 3.14 | (0.38, 0.4) | 0.003 |
|  | LEV-1 | 30.43 | (6.35, 7.08) | 0.001 |
|  | LEV-2 | 5.25 | (1.92, 2.33) | 0.016 |
| *fmtB* | CIP-1 | 8.82 | (2.35, 2.71) | 0.005 |
|  | CIP-2 | 4.26 | (0.49, 0.52) | 0.002 |
|  | OFL-1 | 7.10 | (2.28, 2.7) | 0.009 |
|  | OFL-2 | 3.37 | (0.32, 0.33) | 0.002 |
|  | LEV-1 | 24.66 | (4.88, 5.41) | 0.001 |
|  | LEV-2 | 7.28 | (1.78, 2.02) | 0.005 |
| *Gene encoding hypothetical protein* | CIP-1 | 6.69 | (2.73, 3.48) | 0.017 |
|  | CIP-2 | 2.77 | (0.54, 0.6) | 0.011 |
|  | OFL-1 | 5.65 | (0.58, 0.61) | 0.001 |
|  | OFL-2 | 3.14 | (0.71, 0.79) | 0.012 |
|  | LEV-1 | 25.88 | (7.96, 9.27) | 0.003 |
|  | LEV-2 | 7.51 | (3.07, 3.81) | 0.014 |

**^a^** p-value was obtained from analysis of one-tail Student’s t-test with a 95% confidence.
