# Supplementary material for: Genomic alterations involved in fluoroquinolone resistance development in Staphylococcus aureus
Source: PLoS One. 2023 Jul 26;18(7):e0287973. doi: 10.1371/journal.pone.0287973 (PMC10370734; doi:10.1371/journal.pone.0287973)
Supplement: S1 File — (DOCX) [file pone.0287973.s005.docx]

**MIC values of *S. aureus* during sub-MIC exposure to FQs including ciprofloxacin, ofloxacin, or levofloxacin.**

|  | **Day** | **Ciprofloxacin** | **Ofloxacin** | **Levofloxacin** |
| --- | --- | --- | --- | --- |
|  |  | **MIC (mg/L)** | | |
| Antibiotic exposure | 0 | 0.25 | 0.25 | 0.125 |
|  | 1 | 0.25 | 0.5 | 0.25 |
|  | 2 | 0.5 | 0.5 | 0.25 |
|  | 3 | 1 | 1 | 0.5 |
|  | 4 | 2 | 2 | 0.5 |
|  | 5 | 4 | 2 | 0.5 |
|  | 6 | 8 | 2 | 1 |
|  | 7 | 8 | 4 | 2 |
|  | 8 | 16 | 4 | 2 |
|  | 9 | 16 | 8 | 2 |
|  | 10 | 16 | 8 | 4 |
|  | 11 | 16 | 8 | 4 |
|  | 12 | 16 | 8 | 4 |
| Antibiotic-free culture | R1 | 16 | 8 | 4 |
|  | R2 | 16 | 8 | 4 |
|  | R3 | 8 | 8 | 4 |
|  | R4 | 8 | 8 | 4 |
|  | R5 | 8 | 8 | 4 |
|  | R6 | 8 | 4 | 4 |
|  | R7 | 4 | 4 | 4 |
|  | R8 | 4 | 4 | 2 |
|  | R9 | 4 | 4 | 2 |
|  | R10 | 4 | 4 | 2 |

**The transcriptional values (Ct) of *sigB* were normalized to that of the *16S rRNA* transcripts to obtain a ∆Ct.**

REP1

| *S. aureus* Strains | Ct value | | ∆ Ct | ∆ ∆ Ct | Fold Change |
| --- | --- | --- | --- | --- | --- |
|  | *16S rRNA* | *sigB* |  |  |  |
| ATCC 29213 | 12.16 | 18.22 | 6.06 |  |  |
| CIP-1 | 11.78 | 17.09 | 5.32 | -0.74 | 1.67 |
| CIP-2 | 10.75 | 16.59 | 5.84 | -0.22 | 1.17 |
| OFL-1 | 10.82 | 16.31 | 5.48 | -0.58 | 1.49 |
| OFL-2 | 10.66 | 18.02 | 7.36 | 1.30 | 0.41 |
| LEV-1 | 10.89 | 15.77 | 4.88 | -1.18 | 2.26 |
| LEV-2 | 10.28 | 16.98 | 6.70 | 0.64 | 0.64 |

REP2

| *S. aureus* Strains | Ct value | | ∆ Ct | ∆ ∆ Ct | Fold Change |
| --- | --- | --- | --- | --- | --- |
|  | *16S rRNA* | *sigB* |  |  |  |
| ATCC 29213 | 12.19 | 18.13 | 5.95 |  |  |
| CIP-1 | 11.78 | 17.01 | 5.23 | -0.72 | 1.65 |
| CIP-2 | 10.69 | 16.45 | 5.76 | -0.19 | 1.14 |
| OFL-1 | 10.62 | 15.79 | 5.18 | -0.77 | 1.71 |
| OFL-2 | 10.31 | 17.89 | 7.58 | 1.63 | 0.32 |
| LEV-1 | 10.37 | 15.74 | 5.37 | -0.57 | 1.49 |
| LEV-2 | 9.60 | 16.99 | 7.39 | 1.44 | 0.37 |

REP3

| *S. aureus* Strains | Ct value | | ∆ Ct | ∆ ∆ Ct | Fold Change |
| --- | --- | --- | --- | --- | --- |
|  | *16S rRNA* | *sigB* |  |  |  |
| ATCC 29213 | 12.02 | 18.13 | 6.11 |  |  |
| CIP-1 | 11.66 | 17.01 | 5.35 | -0.76 | 1.69 |
| CIP-2 | 10.71 | 16.57 | 5.86 | -0.25 | 1.19 |
| OFL-1 | 10.92 | 16.66 | 5.73 | -0.38 | 1.30 |
| OFL-2 | 10.91 | 17.98 | 7.07 | 0.96 | 0.51 |
| LEV-1 | 11.31 | 15.65 | 4.34 | -1.77 | 3.42 |
| LEV-2 | 10.32 | 16.81 | 6.49 | 0.38 | 0.77 |

**The transcriptional values (Ct) of *sigS* were normalized to that of the *16S rRNA* transcripts to obtain a ∆Ct**.

REP1

| *S. aureus* Strains | Ct value | | ∆ Ct | ∆ ∆ Ct | Fold Change |
| --- | --- | --- | --- | --- | --- |
|  | *16S rRNA* | *sigS* |  |  |  |
| ATCC 29213 | 12.16 | 19.72 | 7.56 |  |  |
| CIP-1 | 11.78 | 17.96 | 6.19 | -1.38 | 2.60 |
| CIP-2 | 10.75 | 17.55 | 6.80 | -0.77 | 1.70 |
| OFL-1 | 10.82 | 17.44 | 6.62 | -0.94 | 1.92 |
| OFL-2 | 10.66 | 19.08 | 8.42 | 0.85 | 0.55 |
| LEV-1 | 10.89 | 16.55 | 5.66 | -1.90 | 3.74 |
| LEV-2 | 10.28 | 18.06 | 7.77 | 0.21 | 0.86 |

REP2

| *S. aureus* Strains | Ct value | | ∆ Ct | ∆ ∆ Ct | Fold Change |
| --- | --- | --- | --- | --- | --- |
|  | *16S rRNA* | *sigS* |  |  |  |
| ATCC 29213 | 12.19 | 19.66 | 7.47 |  |  |
| CIP-1 | 11.78 | 17.60 | 5.82 | -1.66 | 3.15 |
| CIP-2 | 10.69 | 17.27 | 6.58 | -0.89 | 1.85 |
| OFL-1 | 10.62 | 17.22 | 6.61 | -0.87 | 1.82 |
| OFL-2 | 10.31 | 18.70 | 8.39 | 0.92 | 0.53 |
| LEV-1 | 10.37 | 16.32 | 5.95 | -1.52 | 2.87 |
| LEV-2 | 9.60 | 17.75 | 8.15 | 0.67 | 0.63 |

REP3

| *S. aureus* Strains | Ct value | | ∆ Ct | ∆ ∆ Ct | Fold Change |
| --- | --- | --- | --- | --- | --- |
|  | *16S rRNA* | *sigS* |  |  |  |
| ATCC 29213 | 12.02 | 19.59 | 7.58 |  |  |
| CIP-1 | 11.66 | 18.15 | 6.49 | -1.08 | 2.12 |
| CIP-2 | 10.71 | 17.66 | 6.95 | -0.63 | 1.55 |
| OFL-1 | 10.92 | 17.49 | 6.57 | -1.01 | 2.01 |
| OFL-2 | 10.91 | 19.26 | 8.36 | 0.78 | 0.58 |
| LEV-1 | 11.31 | 16.62 | 5.31 | -2.26 | 4.80 |
| LEV-2 | 10.32 | 18.18 | 7.86 | 0.29 | 0.82 |

**The transcriptional values (Ct) of *mgrA* were normalized to that of the *16S rRNA* transcripts to obtain a ∆Ct.**

REP1

| *S. aureus* Strains | Ct value | | ∆ Ct | ∆ ∆ Ct | Fold Change |
| --- | --- | --- | --- | --- | --- |
|  | *16S rRNA* | *mgrA* |  |  |  |
| ATCC 29213 | 11.99 | 18.89 | 6.90 |  |  |
| CIP-1 | 12.62 | 18.07 | 5.44 | -1.45 | 2.74 |
| CIP-2 | 11.70 | 17.68 | 5.98 | -0.92 | 1.89 |
| OFL-1 | 12.81 | 18.15 | 5.34 | -1.56 | 2.95 |
| OFL-2 | 13.40 | 19.23 | 5.83 | -1.07 | 2.10 |
| LEV-1 | 13.38 | 16.76 | 3.39 | -3.51 | 11.42 |
| LEV-2 | 13.77 | 18.13 | 4.36 | -2.54 | 5.81 |

REP2

| *S. aureus* Strains | Ct value | | ∆ Ct | ∆ ∆ Ct | Fold Change |
| --- | --- | --- | --- | --- | --- |
|  | *16S rRNA* | *mgrA* |  |  |  |
| ATCC 29213 | 7.17 | 18.67 | 11.50 |  |  |
| CIP-1 | 9.07 | 18.11 | 9.05 | -2.45 | 5.48 |
| CIP-2 | 7.48 | 17.57 | 10.09 | -1.41 | 2.66 |
| OFL-1 | 8.45 | 17.93 | 9.48 | -2.02 | 4.06 |
| OFL-2 | 8.81 | 19.17 | 10.36 | -1.14 | 2.21 |
| LEV-1 | 9.24 | 17.29 | 8.05 | -3.45 | 10.95 |
| LEV-2 | 8.67 | 17.91 | 9.24 | -2.26 | 4.80 |

REP3

| *S. aureus* Strains | Ct value | | ∆ Ct | ∆ ∆ Ct | Fold Change |
| --- | --- | --- | --- | --- | --- |
|  | *16S rRNA* | *mgrA* |  |  |  |
| ATCC 29213 | 10.12 | 21.84 | 11.72 |  |  |
| CIP-1 | 12.17 | 21.48 | 9.31 | -2.41 | 5.30 |
| CIP-2 | 10.63 | 21.04 | 10.41 | -1.31 | 2.48 |
| OFL-1 | 11.07 | 21.27 | 10.20 | -1.52 | 2.87 |
| OFL-2 | 12.03 | 22.09 | 10.06 | -1.65 | 3.15 |
| LEV-1 | 12.48 | 20.24 | 7.76 | -3.96 | 15.51 |
| LEV-2 | 11.40 | 21.30 | 9.90 | -1.81 | 3.52 |

**The transcriptional values (Ct) of *norA* were normalized to that of the *16S rRNA* transcripts to obtain a ∆Ct.**

REP1

| *S. aureus* Strains | Ct value | | ∆ Ct | ∆ ∆ Ct | Fold Change |
| --- | --- | --- | --- | --- | --- |
|  | *16S rRNA* | *norA* |  |  |  |
| ATCC 29213 | 11.99 | 20.09 | 8.10 |  |  |
| CIP-1 | 12.62 | 15.43 | 2.81 | -5.29 | 39.23 |
| CIP-2 | 11.70 | 14.35 | 2.65 | -5.45 | 43.68 |
| OFL-1 | 12.81 | 18.82 | 6.01 | -2.09 | 4.27 |
| OFL-2 | 13.40 | 19.99 | 6.60 | -1.50 | 2.84 |
| LEV-1 | 13.38 | 18.01 | 4.64 | -3.46 | 11.04 |
| LEV-2 | 13.77 | 19.20 | 5.43 | -2.67 | 6.37 |

REP2

| *S. aureus* Strains | Ct value | | ∆ Ct | ∆ ∆ Ct | Fold Change |
| --- | --- | --- | --- | --- | --- |
|  | *16S rRNA* | *norA* |  |  |  |
| ATCC 29213 | 7.17 | 20.73 | 13.56 |  |  |
| CIP-1 | 9.07 | 16.08 | 7.02 | -6.54 | 93.29 |
| CIP-2 | 7.48 | 14.95 | 7.48 | -6.08 | 67.84 |
| OFL-1 | 8.45 | 18.81 | 10.36 | -3.20 | 9.16 |
| OFL-2 | 8.81 | 20.45 | 11.63 | -1.93 | 3.80 |
| LEV-1 | 9.24 | 18.25 | 9.01 | -4.55 | 23.40 |
| LEV-2 | 8.67 | 19.57 | 10.89 | -2.67 | 6.34 |

REP3

| *S. aureus* Strains | Ct value | | ∆ Ct | ∆ ∆ Ct | Fold Change |
| --- | --- | --- | --- | --- | --- |
|  | *16S rRNA* | *norA* |  |  |  |
| ATCC 29213 (Pos) | 9.28 | 22.18 | 12.90 |  |  |
| CIP-1 | 11.07 | 18.12 | 7.05 | -5.85 | 57.51 |
| CIP-2 | 10.14 | 16.72 | 6.58 | -6.31 | 79.51 |
| OFL-1 | 11.09 | 21.10 | 10.00 | -2.89 | 7.42 |
| OFL-2 | 10.95 | 22.46 | 11.50 | -1.39 | 2.63 |
| LEV-1 | 11.19 | 20.17 | 8.98 | -3.91 | 15.06 |
| LEV-2 | 10.79 | 21.86 | 11.07 | -1.82 | 3.54 |

**The transcriptional values (Ct) of *norB* were normalized to that of the *16S rRNA* transcripts to obtain a ∆Ct.**

REP1

| *S. aureus* Strains | Ct value | | ∆ Ct | ∆ ∆ Ct | Fold Change |
| --- | --- | --- | --- | --- | --- |
|  | *16S rRNA* | *norB* |  |  |  |
| ATCC 29213 | 11.99 | 19.62 | 7.63 |  |  |
| CIP-1 | 12.62 | 18.48 | 5.86 | -1.77 | 3.41 |
| CIP-2 | 11.70 | 18.07 | 6.37 | -1.26 | 2.39 |
| OFL-1 | 12.81 | 18.26 | 5.45 | -2.18 | 4.53 |
| OFL-2 | 13.40 | 19.82 | 6.42 | -1.21 | 2.31 |
| LEV-1 | 13.38 | 17.69 | 4.31 | -3.32 | 9.99 |
| LEV-2 | 13.77 | 18.69 | 4.92 | -2.71 | 6.54 |

REP2

| *S. aureus* Strains | Ct value | | ∆ Ct | ∆ ∆ Ct | Fold Change |
| --- | --- | --- | --- | --- | --- |
|  | *16S rRNA* | *norB* |  |  |  |
| ATCC 29213 | 7.17 | 20.34 | 13.17 |  |  |
| CIP-1 | 9.07 | 18.92 | 9.85 | -3.32 | 9.98 |
| CIP-2 | 7.48 | 18.66 | 11.19 | -1.99 | 3.96 |
| OFL-1 | 8.45 | 18.43 | 9.98 | -3.19 | 9.13 |
| OFL-2 | 8.81 | 20.51 | 11.69 | -1.48 | 2.78 |
| LEV-1 | 9.24 | 18.10 | 8.87 | -4.31 | 19.77 |
| LEV-2 | 8.67 | 19.03 | 10.36 | -2.81 | 7.03 |

REP3

| *S. aureus* Strains | Ct value | | ∆ Ct | ∆ ∆ Ct | Fold Change |
| --- | --- | --- | --- | --- | --- |
|  | *16S rRNA* | *norB* |  |  |  |
| ATCC 29213 (Pos) | 10.12 | 22.00 | 11.88 |  |  |
| CIP-1 | 12.17 | 21.19 | 9.02 | -2.85 | 7.23 |
| CIP-2 | 10.63 | 20.93 | 10.30 | -1.58 | 2.99 |
| OFL-1 | 11.07 | 21.08 | 10.01 | -1.87 | 3.65 |
| OFL-2 | 12.03 | 22.72 | 10.69 | -1.19 | 2.27 |
| LEV-1 | 12.48 | 20.25 | 7.77 | -4.11 | 17.23 |
| LEV-2 | 11.40 | 21.32 | 9.93 | -1.95 | 3.86 |

**The transcriptional values (Ct) of *norC* were normalized to that of the *16S rRNA* transcripts to obtain a ∆Ct.**

REP1

| *S. aureus* Strains | Ct value | | ∆ Ct | ∆ ∆ Ct | Fold Change |
| --- | --- | --- | --- | --- | --- |
|  | *16S rRNA* | *norC* |  |  |  |
| ATCC 29213 | 11.99 | 19.46 | 7.46 |  |  |
| CIP-1 | 12.62 | 17.79 | 5.16 | -2.30 | 4.92 |
| CIP-2 | 11.70 | 17.41 | 5.71 | -1.75 | 3.37 |
| OFL-1 | 12.81 | 17.96 | 5.15 | -2.31 | 4.97 |
| OFL-2 | 13.40 | 19.01 | 5.61 | -1.85 | 3.61 |
| LEV-1 | 13.38 | 16.74 | 3.36 | -4.10 | 17.19 |
| LEV-2 | 13.77 | 18.09 | 4.32 | -3.14 | 8.83 |

REP2

| *S. aureus* Strains | Ct value | | ∆ Ct | ∆ ∆ Ct | Fold Change |
| --- | --- | --- | --- | --- | --- |
|  | *16S rRNA* | *norC* |  |  |  |
| ATCC 29213 | 7.17 | 19.32 | 12.15 |  |  |
| CIP-1 | 9.07 | 18.08 | 9.01 | -3.14 | 8.84 |
| CIP-2 | 7.48 | 17.93 | 10.45 | -1.70 | 3.25 |
| OFL-1 | 8.45 | 18.13 | 9.68 | -2.48 | 5.57 |
| OFL-2 | 8.81 | 19.43 | 10.61 | -1.54 | 2.91 |
| LEV-1 | 9.24 | 17.12 | 7.88 | -4.28 | 19.39 |
| LEV-2 | 8.67 | 18.24 | 9.57 | -2.59 | 6.00 |

REP3

| *S. aureus* Strains | Ct value | | ∆ Ct | ∆ ∆ Ct | Fold Change |
| --- | --- | --- | --- | --- | --- |
|  | *16S rRNA* | *norC* |  |  |  |
| ATCC 29213 | 10.12 | 21.73 | 11.61 |  |  |
| CIP-1 | 12.17 | 21.02 | 8.86 | -2.75 | 6.73 |
| CIP-2 | 10.63 | 20.56 | 9.93 | -1.68 | 3.21 |
| OFL-1 | 11.07 | 20.96 | 9.89 | -1.72 | 3.29 |
| OFL-2 | 12.03 | 22.38 | 10.35 | -1.25 | 2.38 |
| LEV-1 | 12.48 | 19.46 | 6.98 | -4.63 | 24.70 |
| LEV-2 | 11.40 | 20.95 | 9.55 | -2.06 | 4.16 |

**The transcriptional values (Ct) of *rimI* were normalized to that of the *16S rRNA* transcripts to obtain a ∆Ct.**

REP1

| *S. aureus* Strains | Ct value | | ∆ Ct | ∆ ∆ Ct | Fold Change |
| --- | --- | --- | --- | --- | --- |
|  | *16S rRNA* | *rimI* |  |  |  |
| ATCC 29213 | 7.17 | 19.43 | 12.26 |  |  |
| CIP-1 | 9.07 | 18.19 | 9.12 | -3.14 | 8.82 |
| CIP-2 | 7.48 | 17.98 | 10.50 | -1.76 | 3.39 |
| OFL-1 | 8.45 | 18.19 | 9.74 | -2.52 | 5.75 |
| OFL-2 | 8.81 | 19.40 | 10.58 | -1.68 | 3.21 |
| LEV-1 | 9.24 | 16.49 | 7.25 | -5.02 | 32.37 |
| LEV-2 | 8.67 | 18.08 | 9.41 | -2.86 | 7.25 |

REP2

| *S. aureus* Strains | Ct value | | ∆ Ct | ∆ ∆ Ct | Fold Change |
| --- | --- | --- | --- | --- | --- |
|  | *16S rRNA* | *rimI* |  |  |  |
| ATCC 29213 | 10.12 | 21.85 | 11.73 |  |  |
| CIP-1 | 12.17 | 20.94 | 8.78 | -2.95 | 7.72 |
| CIP-2 | 10.63 | 20.46 | 9.82 | -1.90 | 3.74 |
| OFL-1 | 11.07 | 20.56 | 9.49 | -2.23 | 4.70 |
| OFL-2 | 12.03 | 21.98 | 9.95 | -1.78 | 3.43 |
| LEV-1 | 12.48 | 19.08 | 6.60 | -5.12 | 34.84 |
| LEV-2 | 11.40 | 20.80 | 9.40 | -2.32 | 5.00 |

REP3

| *S. aureus* Strains | Ct value | | ∆ Ct | ∆ ∆ Ct | Fold Change |
| --- | --- | --- | --- | --- | --- |
|  | *16S rRNA* | *rimI* |  |  |  |
| ATCC 29213 | 9.28 | 21.07 | 11.79 |  |  |
| CIP-1 | 11.07 | 20.05 | 8.98 | -2.81 | 7.02 |
| CIP-2 | 10.14 | 19.62 | 9.48 | -2.30 | 4.93 |
| OFL-1 | 11.09 | 19.68 | 8.59 | -3.20 | 9.16 |
| OFL-2 | 10.95 | 21.27 | 10.32 | -1.47 | 2.77 |
| LEV-1 | 11.19 | 18.39 | 7.20 | -4.59 | 24.07 |
| LEV-2 | 10.79 | 20.76 | 9.98 | -1.81 | 3.51 |

**The transcriptional values (Ct) of *fmtB* were normalized to that of the *16S rRNA* transcripts to obtain a ∆Ct.**

REP1

| *S. aureus* Strains | Ct value | | ∆ Ct | ∆ ∆ Ct | Fold Change |
| --- | --- | --- | --- | --- | --- |
|  | *16S rRNA* | *fmtB* |  |  |  |
| ATCC 29213 | 7.17 | 19.95 | 12.78 |  |  |
| CIP-1 | 9.07 | 18.49 | 9.42 | -3.36 | 10.28 |
| CIP-2 | 7.48 | 18.12 | 10.64 | -2.14 | 4.42 |
| OFL-1 | 8.45 | 18.42 | 9.97 | -2.82 | 7.04 |
| OFL-2 | 8.81 | 19.78 | 10.97 | -1.81 | 3.52 |
| LEV-1 | 9.24 | 17.31 | 8.08 | -4.71 | 26.14 |
| LEV-2 | 8.67 | 18.32 | 9.64 | -3.14 | 8.81 |

REP2

| *S. aureus* Strains | Ct value | | ∆ Ct | ∆ ∆ Ct | Fold Change |
| --- | --- | --- | --- | --- | --- |
|  | *16S rRNA* | *fmtB* |  |  |  |
| ATCC 29213 | 10.12 | 22.44 | 12.32 |  |  |
| CIP-1 | 12.17 | 21.20 | 9.04 | -3.28 | 9.73 |
| CIP-2 | 10.63 | 21.04 | 10.41 | -1.91 | 3.77 |
| OFL-1 | 11.07 | 21.08 | 10.01 | -2.31 | 4.97 |
| OFL-2 | 12.03 | 22.52 | 10.50 | -1.83 | 3.54 |
| LEV-1 | 12.48 | 19.99 | 7.51 | -4.81 | 28.07 |
| LEV-2 | 11.40 | 21.24 | 9.84 | -2.48 | 5.56 |

REP3

| *S. aureus* Strains | Ct value | | ∆ Ct | ∆ ∆ Ct | Fold Change |
| --- | --- | --- | --- | --- | --- |
|  | *16S rRNA* | *fmtB* |  |  |  |
| ATCC 29213 | 9.28 | 22.03 | 12.75 |  |  |
| CIP-1 | 11.07 | 21.14 | 10.06 | -2.69 | 6.44 |
| CIP-2 | 10.14 | 20.69 | 10.55 | -2.20 | 4.60 |
| OFL-1 | 11.09 | 20.63 | 9.53 | -3.22 | 9.30 |
| OFL-2 | 10.95 | 22.10 | 11.15 | -1.61 | 3.04 |
| LEV-1 | 11.19 | 19.64 | 8.45 | -4.31 | 19.77 |
| LEV-2 | 10.79 | 20.64 | 9.85 | -2.90 | 7.46 |

**The transcriptional values (Ct) of gene encoding hypothetical protein were normalized to that of the *16S rRNA* transcripts to obtain a ∆Ct.**

REP1

| *S. aureus* Strains | Ct value | | ∆ Ct | ∆ ∆ Ct | Fold Change |
| --- | --- | --- | --- | --- | --- |
|  | *16S rRNA* | *Hypothetical protein* |  |  |  |
| ATCC 29213 | 11.99 | 19.76 | 7.77 |  |  |
| CIP1 | 12.62 | 18.42 | 5.80 | -1.97 | 3.93 |
| CIP2 | 11.70 | 18.28 | 6.58 | -1.19 | 2.28 |
| OFL1 | 12.81 | 18.24 | 5.43 | -2.34 | 5.06 |
| OFL2 | 13.40 | 19.83 | 6.43 | -1.34 | 2.53 |
| LEV1 | 13.38 | 16.83 | 3.46 | -4.31 | 19.89 |
| LEV2 | 13.77 | 18.09 | 4.31 | -3.46 | 10.97 |

REP2

| *S. aureus* Strains | Ct value | | ∆ Ct | ∆ ∆ Ct | Fold Change |
| --- | --- | --- | --- | --- | --- |
|  | *16S rRNA* | *Hypothetical protein* |  |  |  |
| ATCC 29213 | 7.17 | 19.91 | 12.74 |  |  |
| CIP-1 | 9.07 | 18.75 | 9.68 | -3.06 | 8.35 |
| CIP-2 | 7.48 | 18.77 | 11.29 | -1.46 | 2.74 |
| OFL-1 | 8.45 | 18.63 | 10.18 | -2.56 | 5.90 |
| OFL-2 | 8.81 | 19.97 | 11.16 | -1.59 | 3.01 |
| LEV-1 | 9.24 | 17.48 | 8.24 | -4.50 | 22.65 |
| LEV-2 | 8.67 | 18.66 | 9.99 | -2.75 | 6.74 |

REP3

| *S. aureus* Strains | Ct value | | ∆ Ct | ∆ ∆ Ct | Fold Change |
| --- | --- | --- | --- | --- | --- |
|  | *16S rRNA* | *Hypothetical protein* |  |  |  |
| ATCC 29213 | 10.12 | 22.23 | 12.11 |  |  |
| CIP-1 | 12.17 | 21.31 | 9.15 | -2.96 | 7.78 |
| CIP-2 | 10.63 | 21.02 | 10.39 | -1.72 | 3.29 |
| OFL-1 | 11.07 | 20.60 | 9.53 | -2.58 | 5.98 |
| OFL-2 | 12.03 | 22.18 | 10.16 | -1.95 | 3.87 |
| LEV-1 | 12.48 | 19.45 | 6.97 | -5.13 | 35.10 |
| LEV-2 | 11.40 | 21.23 | 9.84 | -2.27 | 4.83 |
