# Supplementary material for: Genomic alterations involved in fluoroquinolone resistance development in Staphylococcus aureus
Source: PLoS One. 2023 Jul 26;18(7):e0287973. doi: 10.1371/journal.pone.0287973 (PMC10370734; doi:10.1371/journal.pone.0287973)
Supplement: Attachment — Submitted filename: Response to Reviewers.docx [file pone.0287973.s006.docx]

Dear Reviewers,

Thank you very much for your comments!

We have taken your comments seriously and revised markedly our manuscript. All the revised parts of the manuscript have been highlighted yellow in the re-submitted version.

Below is the point-to-point response to your comments!

Thank you very much again for your time and valuable comments!

| **COMMENTS OF REVIEWER** | **RESPONSE TO REVIEWER** |
| --- | --- |
| **ABSTRACT** | |
| Abstract is too long. It must be not more than 250 words. Please add background/problem statement and objectives. The abstract did not reflect the main findings of the research. | The abstract has been revised: shortened to 250 words;  The problem statement and objectives were clarified. The main findings were shown. |
| **INTRODUCTION** | |
| 1. It is good in storyline however more information need to be addressed what cause the resistance to the antibiotic and why mutation occurred or what trigger this mutation in normal circumstances not mentioned. | The information about how bacteria become resistant to antibiotics and the emergence of mutation during antibiotic-resistant development were added in **paragraph 2, 3, 4, 5** of **Introduction** section. |
| 2. Mention the type of mutations and type of important drug efflux pumps of *S. aureus* belonging families which contributes to resistance to fluoroquinolones. Also introduction must be explain the gene encode for multidrug resistance efflux pump. | The multidrug-resistant efflux system encoded by *norA, norB*, and *norC* which contribute to FQ resistance was mentioned in **paragraph 5** of **Introduction** section. |
| 3. Why choose (*grlA/B*) not (*parC/parE*)? this is not address in Introduction section as well. What's the total length of *grlA/B*? | In *S. aureus* and some species, genes encoding for topoisomerase IV are named *grlA* and *grlB* instead of *parC/E*. The explanation and the emergence of FQ resistance by FQ target alterations were also mentioned in **paragraph 4** of **Introduction** section |
| **METHODOLOGY** | |
| 1. Comprehensive and adequate, but you can add the section of statistical analysis in methods; the authors may use correlation to be more informative | The statistical analysis section was added in the **last paragraph** of **Method** section. The statistical analysis describes:   - RT-qPCR data collection. - Fold change and confident level calculation using MS Excel. - Statistical analysis for significant differences. |
| 2. Why used *S. aureus* ATCC 29213 and expose it FQ to obtain resistance strains? you could isolate resistance strains to FQ from different clinical settings what the purpose? | The explanation was added in the **paragraph 2** (for the complication of AMR in clinical isolates), **paragraph 6** of the introduction and first sentence of the method part (for the use of ATCC 29213). |
| **RESULTS** | |
| 1. In line 128 Comparison between strains before exposure to treatment with FQ and after insufficient should be reformulated in a more understandable way | Line 128: Fig 1’s legend was now rewritten into “MIC values of *S. aureus* during sub-MIC exposure to FQs including ciprofloxacin (CIP) (A), ofloxacin (OFL) (B), or levofloxacin (LEV) (C). The initial *S. aureus* was exposed to CIP, OFL, or LEV for 12 days to obtain FQ-resistant *S. aureus* strains (CIP-1, OFL-1, and LEV-1). These exposed *S. aureus* strains were then continuously sub-cultured for another 10 days in an antibiotic-free medium to obtain CIP-2, OFL-2, and LEV-2 with the daily examination of MICs values was evaluated. Day 0: MIC; day 1-12: 12 FQ exposed days; R1-R10: 10 days in antibiotic-free medium.”  “*S. aureus*-1” and “*S. aureus*-2” were added in text from line 159 to 164 to make sentences clearer. |
| 2. Table 2, the title and the content are not well described in the paper, please change the title or add some sentence or reference in the paper. | The title of Table 2 was changed to “Antibiotic susceptibility profile of *S. aureus* ATCC 29213 (initial strain), *S. aureus*-1 (CIP-1, OFL-1, and LEV-1) and *S. aureus*-2 (CIP-2, OFL-2, and LEV-2).” |
| 3. Why did the authors focus on *grlA/B* not *parC* and mutation in this gene first step in the resistance to most fluoroquinolones. | The reasons why this study mentioned *grlA/B* instead of *parC* was explained in **paragraph 4** of **Introduction** section |
| 4. Fig 1 hardly to read. Only fig quality is not enough? | The MIC values of *S. aureus* during sub-MIC exposure to FQs is now presented in three separate charts corresponding to three used antibiotics ciprofloxacin (CIP) (A), ofloxacin (OFL) (B), or levofloxacin (LEV) (C) for clarity. |
| 5. Quality of images was low? | The quality of images was now improved following the **Figure File Requirements** of PlosOne |
| **DISCUSSION** | |
| 1. Interestingly, we observed the 277 revertance of all mutations in grlA (turning back to the wild-type genotype) without a significant MIC 278 decrease in OFL- and LEV-2 and only a four-time reduction in CIP-revertant strain when both mutations 279 in *grlA* (S80F) and in *gyrB* (R450S) were together disappeared. Moreover, even though OFL-2 kept most 280 of its resistant ability, no mutation in *gyrA, gyrB, grlA*, and *grlB* QRDRs was retained. Our results were 281 similar to the observation in E. faecium of Yoshihiro et al. who proposed that other resistance mechanism(s) 282 such as multidrug resistance efflux pumps might be involved in the development of FQ resistance. How similar"?/ Statistical analysis is needed. | The word “similar” was confusing therefore, the third paragraph (line 304-314) in “**Genomic alterations involved in FQ-resistant development in *S. aureus***” in **Discussion** section was now rewritten to make the idea more understandable. |
| 2. There is a long-established correlation between increased resistance and fitness cost [30]. If the FQ-resistance mechanism imposes a fitness cost, it would be advantageous for bacteria to be able to exhibit the resistance phenotype only when the threat exists. Even if there is no fitness cost, the production of resistance-mediating proteins could impose an unnecessary metabolic burden on the bacteria in an 289 antimicrobial-free environment. Rewrite the sentences in a more understandable way? | The discussions on “**Changes in expression of alternative sigma factors associated with the adaptive response of *S. aureus* under serial FQ exposure”** (line 317-333) were rewritten to include the following points:   - Overview the fuction of sigma factor in stress-adaptive responses in bacteria - The findings in sigma factor expression in this study. - The explanation and the suggestion of this study. |
| **CONCLUSION** | |
| Author must address whether resistance to FQ is an alarming situation or not since the resistances of *S. aureus* to FQ were reported in many other countries as well. | The conclusion has highlighted the role of FQs in infectious treatment and confirmed the danger of FQ-resistant development for staph infection treatment all over the world. |
| **REFERENCES** | |
| Many references are OLD. Please include the latest especially on Introduction and Discussions. Thus, we prefer to use the update and following references:- 1-Hussein, RA, Al-Ouqaili MTS, Majeed YH. (2022). Detection of clarithromycin resistance and 23SrRNA point mutations in clinical isolates of Helicobacter pylori isolates: Phenotypic and molecular methods, Saudi Journal of Biological Sciences, 29 (1). 2- Al-Ouqaili, M.T.S., Al-Kubaisy, S.H.M., Al-Ani, N.F.I. Biofilm antimicrobial susceptibility pattern for selected antimicrobial agents against planktonic and sessile cells of clinical isolates of staphylococci using MICs, BICs and MBECs. Asian Journal of Pharmaceutics, volume 12, Issue 4, October-December 2018, Pages S1375-S1383. 3- Al-Qaysi, A. K., Al-Ouqaili, . M. T. & Al-Meani, . S. A. (2020). Ciprofloxacin- and gentamicin-mediated inhibition of Pseudomonas aeruginosa biofilms is enhanced when combined the volatile oil from Eucalyptus camaldulensis. SRP, 11 (7), 98-105. 4-Chinemerem Nwobodo D, Ugwu MC, Oliseloke Anie C, Al-Ouqaili MTS, Chinedu Ikem J, Victor Chigozie U, Saki M. Antibiotic resistance: The challenges and some emerging strategies for tackling a global menace. J Clin Lab Anal. 2022 Sep;36(9):e24655. doi: 10.1002/jcla.24655. Epub 2022 Aug 10. PMID: 35949048; PMCID: PMC9459344. | The references published before 2000 was replaced by updated ones.  The 2nd, 3rd and 4th references suggested by reviewer were cited in this study. |
